# ESBL- and Carbapenemase-Producing *Enterobacteriaceae* in Patients with Bacteremia, Yangon, Myanmar, 2014

**DOI:** 10.3201/eid2305.161100

**Published:** 2017-05

**Authors:** Tin O. Myat, Rachel F. Hannaway, Khwar N. Zin, Wah W. Htike, Kyu K. Win, John A. Crump, David R. Murdoch, James E. Ussher

**Affiliations:** University of Medicine 1, Yangon, Myanmar (T.O. Myat, W.W. Htike, K.K. Win);; University of Otago, Dunedin, New Zealand (R.F. Hannaway, J.A. Crump, J.E. Ussher);; Yangon General Hospital, Yangon (K.N. Zin);; University of Otago, Christchurch, New Zealand (D.R. Murdoch);; Southern Community Laboratories, Dunedin Hospital, Dunedin (J.E. Ussher)

**Keywords:** β-lactamase, extended-spectrum β-lactamase, New Delhi **metallo**-β-lactamase, ESBL, NDM, CTX-M, Enterobacteriaceae, bacteria, Yangon, Myanmar, carbapenem, carbapenemase, sepsis, antimicrobial resistance

## Abstract

Among 42 gram-negative bloodstream isolates from inpatients in 3 hospitals in Yangon, Myanmar, admitted during July–December 2014, 16 (38%) were extended-spectrum β-lactamase–producing *Enterobacteriaceae* and 6 (14%) produced carbapenemase. The high prevalence of multidrug-resistant gram-negative bacteria raises concerns about the empiric treatment of patients with sepsis in Yangon.

Infections with extended-spectrum β-lactamase (ESBL)–producing gram-negative bacteria and carbapenem-resistant *Enterobacteriaceae* (CRE) have been reported worldwide (*1*). Little is known about the occurrence of ESBL-producing and CRE bacteria in Yangon, Myanmar. Therefore, we characterized 42 gram-negative organisms isolated from routine blood cultures from adult inpatients in Yangon.

All bacteria had been isolated at the microbiology laboratories of 3 hospitals in Yangon during July–December 2014. During the study period, 592 blood cultures were processed, 536 from Yangon General Hospital (YGH) and 56 from 2 private hospitals. YGH is a 2,000-bed tertiary referral and teaching hospital in Yangon, providing free hospital care to civilians. The 2 private hospitals have 350 and 100 beds and provide secondary-level medical and surgical services to paying patients. 

Of the 592 blood cultures, 42 (7.8%) yielded gram-negative bacteria, 28 (67%) from YGH and 14 (33%) from the 2 private hospitals. No clinical information was available about the patients from whom the cultures were taken. The identity and antimicrobial drug susceptibility of isolates were confirmed at Southern Community Laboratories (Dunedin, New Zealand) by matrix-assisted laser desorption/ionization time-of-flight mass spectrometry (Microflex LT; Bruker Daltonics, Billerica, MA, USA), disc diffusion testing using the Clinical and Laboratory Standards Institute method (*2*), and the Phoenix Automated Microbiology System (Bruker Daltonics) (panel NMIC/ID-95).

We conducted phenotypic confirmation of ESBL production on cefpodoxime-resistant isolates using cefotaxime and ceftazidime with and without clavulanic acid and that of carbapenmase production on meropenem-resistant isolates by modified Hodge test according to Clinical and Laboratory Standards Institute criteria (*2*). We performed PCR for β-lactamase genes on all ESBL- and potential carbapenemase-producing organisms (*3**,**4*). We conducted bidirectional Sanger sequencing of amplicons and identified DNA sequences by BLAST (https://blast.ncbi.nlm.nih.gov/Blast.cgi) and by comparison with known β-lactamase gene sequences.

Of the 42 isolates, 34 (81%) were *Enterobacteriaceae* (20 *Escherichia coli*, 7 *Klebsiella pneumoniae*, 6 *Salmonella enterica*, and 1 *Enterobacter cloacae*) and 8 (19.0%) were nonfermenting gram-negative bacilli. Of the *Enterobacteriaceae*, 20 (59%) were multidrug resistant (MDR), with resistance to >3 classes of antimicrobial drugs, and 7 (21%) were extensively drug resistant, with susceptibility to <2 classes of antimicrobial drugs (*5*). All MDR *Enterobacteriaceae* were susceptible to polymyxin (Table). Phenotypic testing suggested the presence of an ESBL in 16 (38%) and a carbapenemase in 6 (14%) of all gram-negative isolates. Molecular analysis showed that 13 (81%) ESBL-producing isolates contained a group 1 CTX-M gene; all were confirmed as CTX-M-15 by sequencing. Carbapenemase-producing isolates, including 3 *E. coli* and 3 *K. pneumoniae*, contained the New Delhi **metallo**-β-lactamase (NDM) gene, sequenced as NDM-4 in 5 (83%) and NDM-7 in 1 (17%).

**Table:** Antimicrobial drug susceptibility of *Enterobacteriaceae* and *Acinetobacter* spp. isolated from blood cultures of inpatients from 3 hospitals in Yangon, Myanmar, 2014*

	Organism, no. (%) susceptible				
Agent	*Escherichia coli, *n = 20	*Klebsiella pneumoniae, *n = 7	*Salmonella enterica, *n = 6	*Enterobacter cloacae*, n = 1	*Acinetobacter* spp., n = 3
Ampicillin	2 (10)	0	6 (100)	0	NT
Amoxicillin/clavulanic acid	5 (25)	1 (14)	6 (100)	0	NT
Piperacillin/tazobactam	13 (65)	2 (29)	6 (100)	0	2 (67)
Cefuroxime	5 (25)	1 (14)	NT	0	NT
Ceftriaxone	6 (30)	1 (14)	6 (100)	0	1 (33)
Ceftazidime	6 (30)	1 (14)	6 (100)	0	0
Cefipime	7 (35)	1 (14)	6 (100)	0	2 (67)
Cefoxitin	11 (55)	4 (57)	NT	0	NT
Aztreonam	6 (30)	1 (14)	6 (100)	0	NT
Ertapenem	17 (85)	4 (57)	6 (100)	1 (100)	NT
Imipenem	17 (85)	4 (57)	6 (100)	1 (100)	3 (100)
Meropenem	17 (85)	4 (57)	6 (100)	1 (100)	3 (100)
Gentamicin	14 (70)	2 (29)	NT	1 (100)	2 (67)
Tobramycin	9 (45)	1 (14)	NT	0	3 (100)
Ciprofloxacin	1 (5)	1 (14)	3 (43)	0	2 (67)
Chloramphenicol	11 (55)	2 (29)	6 (100)	0	NT
Colistin†	20 (100)	7 (100)	6 (100)	1 (100)	3 (100)
Nitrofurantoin	16 (80)	1 (14)	NT	0	NT
Trimethoprim/ sulfamethoxazole	2 (10)	2 (29)	6 (100)	0	2 (67)

*NT, not tested. †Because there are no Clinical and Laboratory Standards Institute for susceptibility testing criteria for colistin for *Enterobaceriaceae*, European Committee on Antimicrobial Susceptibility Testing (EUCAST) criteria were used (susceptible if MIC <2 mg/L, per EUCAST criteria version 6.0, 2016; http://www.eucast.org/fileadmin/src/media/PDFs/EUCAST_files/Breakpoint_tables/v_6.0_Breakpoint_table.pdf).

Our study revealed a high proportion of ESBL- and carbapenemase-producing organisms among gram-negative bloodstream isolates during the study period from hospital inpatients in Yangon. Half of *E. coli* isolates and 43% of *K. pneumoniae* isolates produced ESBLs. This finding is consistent with the high proportion of ESBL production reported in isolates from India (>80%), China (>60%), and other Asia and Southeast Asia countries (>30%) (*6*). Carbapenemase production (15% in *E. coli* and 43% in *K. pneumoniae*) in this study was comparable to those previously reported from clinical isolates in India (*7*).

CTX-M-15 ESBL and NDM carbapenemase were the most prevalent mechanisms of resistance to β-lactams in our study. This finding is consistent with the current global dissemination of CTX-M-15 among *E. coli* isolates (*8*). All CRE isolates were NDM-4 or NDM-7. Two previous case reports have indicated the presence of NDM-producing *Enterobacteriaceae* from travelers to Myanmar: 1 NDM-7 (*9*) and 1 NDM-4 (*10*).

Two thirds of all isolates included in this study originated from YGH, the largest public hospital in Myanmar. All CRE isolates and 14 (88%) of 16 ESBL producers were isolated from YGH; this may reflect a higher prevalence of colonization with MDR organisms among patients in YGH or they may be healthcare-associated infections. Of concern, at YGH 11 (73%) of 15 *E. coli* and 6 (100%) of 6 *K. pneumoniae* isolates produced either an ESBL or carbapenemase; among these, 3 (20%) of 15 *E. coli* and 3 (50%) of 6 *K. pneumoniae* isolates were NDM producers.

Whereas all 23 (100%) of the *Enterobacteriaceae* at YGH were susceptible to treatment with colistin, an empiric treatment regimen of meropenem plus gentamicin would have covered only 18 (78%) isolates. This finding highlights the difficulties with designing an effective empiric antimicrobial regimen for patients with suspected gram-negative sepsis in a setting of a high prevalence of antimicrobial resistance, without providing further selective pressure for the spread of CRE and the emergence of colistin resistance.

Our study has limitations. First, clinical data were not prospectively collected, and it was not possible to obtain data retrospectively because of poor recording systems. Second, we cannot be certain that study isolates represent the population of organisms causing gram-negative sepsis in Yangon. However, the high proportion of ESBL- and carbapenemase-producing gram-negative bacteria among bloodstream isolates from hospitalized patients in Yangon raises concern for the treatment of patients with gram-negative sepsis and suggests a need to reduce selective pressure and control the spread of resistant organisms.
